# An accelerated mouse model for atherosclerosis and adipose tissue inflammation

**DOI:** 10.1186/1475-2840-13-23

**Published:** 2014-01-17

**Authors:** Angelika Neuhofer, Bernhard Wernly, Lukas Leitner, Alisina Sarabi, Nicole G Sommer, Günther Staffler, Maximilian Zeyda, Thomas M Stulnig

**Affiliations:** 1 Christian Doppler Laboratory for Cardio-Metabolic Immunotherapy and Clinical Division of Endocrinology and Metabolism, Department of Medicine III, Medical University of Vienna, Vienna, Austria; 2 AFFiRiS AG, Vienna, Austria

**Keywords:** Metabolic syndrome, Diet-induced obesity, Sucrose-enrichment, Adipose tissue inflammation, Insulin resistance, Glucose intolerance, Atherosclerosis, Type 2 diabetes, Cardio-metabolic disease

## Abstract

**Background:**

Obesity and particularly the metabolic syndrome, which is often associated with obesity, combine a major risk for type 2 diabetes and cardiovascular disease. Emerging evidence indicate obesity-associated subclinical inflammation primarily originating from adipose tissue as a common cause for type 2 diabetes and cardiovascular disease. However, a suitable and well-characterized mouse model to simultaneously study obesity-associated metabolic disorders and atherosclerosis is not available yet. Here we established and characterized a murine model combining diet-induced obesity and associated adipose tissue inflammation and metabolic deteriorations as well as atherosclerosis, hence reflecting the human situation of cardio-metabolic disease.

**Methods:**

We compared a common high-fat diet with 0.15% cholesterol (HFC), and a high-fat, high-sucrose diet with 0.15% cholesterol (HFSC) fed to LDL receptor-deficient (LDLR^-/-^) mice. Insulin resistance, glucose tolerance, atherosclerotic lesion formation, hepatic lipid accumulation, and inflammatory gene expression in adipose tissue and liver were assessed.

**Results:**

After 12–16 weeks, LDLR^-/-^ mice fed HFSC or HFC developed significant diet-induced obesity, adipose tissue inflammation, insulin resistance, and impaired glucose tolerance compared to lean controls. Notably, HFSC-fed mice developed significantly higher adipose tissue inflammation in parallel with significantly elevated atherosclerotic lesion area compared to those on HFC. Moreover, LDLR^-/-^ mice on HFSC showed increased insulin resistance and impaired glucose tolerance relative to those on HFC. After prolonged feeding (20 weeks), however, no significant differences in inflammatory and metabolic parameters as well as atherosclerotic lesion formation were detectable any more between LDLR^-/-^ mice fed HFSC or HFC.

**Conclusion:**

The use of high sucrose rather than more complex carbohydrates in high-fat diets significantly accelerates development of obesity-driven metabolic complications and atherosclerotic plaque formation parallel to obesity-induced adipose tissue inflammation in LDLR^-/-^ mice. Hence LDLR^-/-^ mice fed high-fat high-sucrose cholesterol-enriched diet appear to be a suitable and time-saving animal model for cardio-metabolic disease. Moreover our results support the suggested interrelation between adipose tissue inflammation and atherosclerotic plaque formation.

## Background

Obesity is strongly associated with the so-called metabolic syndrome, which comprises a combination of factors conferring risk for type 2 diabetes and cardiovascular disease [1]. Hence in humans, risk for metabolic deterioration and atherosclerosis often occurs in common and is based on insulin resistance and inflammation [2,3]. Inflammation is a common soil of insulin resistance and atherosclerosis underlying type 2 diabetes and cardiovascular disease, respectively. Especially obesity-induced chronic low grade inflammation that primarily originates from the adipose tissue has been shown to play a crucial role in the development of obesity-related diseases [4,5]. In particular, visceral adipose tissue and liver are the primary source and target of circulating inflammatory mediators in obesity-induced inflammation [6]. In both adipose tissue and liver, macrophages, and other immune cells such as T-cells are a main source of inflammatory cytokines such as tumor necrosis factor-α (TNFα), interleukin-6 (IL-6), monocyte chemoattractant protein-1 (MCP-1), and osteopontin [7-9] which drive the low-grade inflammation by mechanisms which need to be elucidated in more detail.

Recently, osteopontin has been identified as one of the key molecules involved in the pathogenesis of atherosclerosis [10-13], as well as type 2 diabetes [14,15]. Moreover, osteopontin plays a role in the pathogenesis of nonalcoholic fatty liver disease (NAFLD) [16,17].

Epidemiological studies emphasize the relationship between sugar consumption e.g. by sugar-sweetened beverages, long-term weight gain and type 2 diabetes mellitus [18,19]. Furthermore, rapidly absorbable carbohydrates such as sucrose leading to high dietary glycemic load may increase type 2 diabetes and cardiovascular risk independently of obesity by provoking inflammation and insulin resistance [20-22]. In addition, fructose also increases blood pressure, dyslipidemia and visceral adiposity [23,24].

In order to reflect the highly prevalent human situation of cardio-metabolic risk and to elucidate mechanisms by which metabolic complications associated with obesity accelerate atherosclerosis in more detail, a well-characterized mouse model is necessary which combines obesity, and insulin resistance with development of significant atherosclerosis. Although a number of mouse strains develop obesity and obesity-associated insulin resistance under certain conditions [25,26] most of these models are resistant to atherosclerosis [27]. On the other hand, both LDL receptor-deficient (LDLR^-/-^) and particularly apolipoprotein E-deficient (ApoE^-/-^) mice display marked atherosclerosis [28,29] but ApoE^-/-^ mice develop lower diet-induced obesity, less profound insulin resistance and adipose tissue inflammation when fed a high-fat diet [30-32].

Hence, LDLR^-/-^ mice seem to be a valuable basis to establish an atherosclerotic mouse model combined with high-fat diet-induced obesity and insulin resistance. However, the impact of a high glycemic load has not been compared to a common high-fat diet in this murine model. In order to develop and standardize a mouse model reflecting the human situation of metabolic syndrome with diet-induced obesity, adipose tissue inflammation promoting insulin resistance and atherosclerosis we investigated the effect of a common high-fat diet and a sucrose-enriched high-fat diet in LDLR^-/-^ mice. In addition, ApoE^-/-^ mice were used as a positive control for atherosclerotic plaque formation.

Here we demonstrate that feeding LDLR^-/-^ mice a high-fat, high-sucrose cholesterol-enriched diet for 16 weeks represents a suitable mouse model for human cardio-metabolic disease. Moreover, our findings suggest an interrelation between increased obesity-associated inflammation and elevated atherosclerotic plaque formation in LDLR^-/-^ mice.

## Methods

### Animals and diet

Male LDLR^-/-^ and ApoE^-/-^ mice, both on a C57BL/6 J background were purchased from Charles River Laboratories (Sulzfeld, Germany). At 9 weeks of age LDLR^-/-^ mice were placed on either a high-fat diet enriched with 0.15% cholesterol (HFC) containing 60 kcal% fat (primarily lard) and 20 kcal% carbohydrates with 6.8 kcal% deriving from sucrose (D01120401; Research Diets Inc., New Brunswick, NJ, USA), a sucrose-enriched high-fat diet with 0.15% cholesterol (HFSC) consisting of 58 kcal% fat (primarily lard) and 28 kcal% carbohydrates (with 17.5 kcal% from sucrose; D09071704, Research Diets Inc), [33] or a low-fat control diet (LF, 10 kcal% fat; D12450B; Research Diets Inc.) for up to 20 weeks. Further details about composition of the used diets are presented as Additional file 1: Table S1. In addition, ApoE^-/-^ mice were fed HFSC for 16 weeks. LDLR^-/-^ mice on LF served as a negative control for adipose tissue inflammation and insulin resistance.

All mice were housed in a specific pathogen-free facility on a 12-hour light/dark cycle with free access to food and water. Food intake and weight gain were monitored throughout the studies. To determine food intake, diets were weighed before and after food change and difference was calculated as estimated food intake. Blood was drawn after a 3-hour fasting period immediately before mice were sacrificed. Gonadal adipose tissue and liver samples were collected and immediately snap frozen in liquid nitrogen. The study protocols were approved by the Austrian Federal Ministry for Science and Research and followed the guidelines on accommodations and care of animals formulated by the European Convention for Protection of Vertebrate Animals Used for Experimental and Other Scientific Purposes.

### Metabolic analyses

Plasma triglyceride and cholesterol concentration was analyzed using an automated analyzer (Falcor 350, A. Menarini Diagnostics, Florence, Italy). Enzyme-linked immunosorbent assay kits were used to determine plasma insulin (Mercodia AB, Uppsala, Sweden) and osteopontin levels (R&D Systems, Minneapolis, MN USA). Homeostasis model assessment of insulin resistance (HOMA-IR) was calculated as an index for insulin resistance [34]. Insulin sensitivity was assessed by insulin tolerance test after a 5-hour fasting period. Briefly, intraperitoneal injection of recombinant human insulin aspart (Novo Nordisk A/S, Denmark) at a dose of 0.75 U/kg body weight was given to HFC- and HFSC-fed LDLR^-/-^ mice and HFSC-fed ApoE^-/-^ mice. Blood glucose concentrations were determined before and 30, 60, 90 and 120 minutes after insulin injection. Glucose tolerance test was performed after a 6-hour fasting period and blood glucose was measured before and 15, 45, 75, 105 and 135 minutes after intraperitoneal injection of 20% glucose (0.75 g/kg body weight).

### FPLC analysis of plasma lipoproteins

To analyze plasma lipoproteins 12 μL of EDTA-treated plasma were injected by autosampler into an Agilent 1200 HPLC instrument equipped with a Superose 6 10/300 gel-filtration FPLC column which separates the intact lipoproteins by size. An in-line assay for total cholesterol (Infinity Cholesterol reagent, Thermo Scientific) was performed at 37°C using a post-column reaction. Reaction products were monitored in real-time at 500 nm and the data analyzed using Agilent Chemstation software.

### Real-time quantitative RT-PCR

Tissue samples were homogenized in TRIzol reagent (Life Technologies, Carlsbad, CA, USA) and RNA was isolated according to the manufacturer’s protocol. One microgram of total RNA was treated with DNase I and transcribed to cDNA using Superscript II and random hexamer primers (all Invitrogen) as described [35]. Gene expression of F4/80 (*Emr1*, Mm00802530_m1), MCP-1 (*Ccl2*, Mm00441242_m1), TNFα (*Tnf*, Mm00443258_m1), IL-6 (*Il6*, Mm00446190_m1), osteopontin (*Spp1*, Mm00436767_m1), adiponectin (*Adipoq*, Mm00456425_m1), fatty acid synthase (*Fasn,* Mm00662319_m1), acetyl-CoA carboxylase (*Acaca,* Mm01304289_m1), stearoyl-CoA desaturase 1 (*Scd1*, Mm00772290_m1), CD3 (*Cd3e*, Mm01179194_m1), CD8 (*Cd8a*, Mm01182108_m1), FoxP3 (*Foxp3*, Mm00475162_m1), CD19 (*Cd19*, Mm00515420_m1), fatty acid binding protein 4 (FABP4; *Fabp4*, Mm00445878_m1), leptin (*Lep*, Mm00434759_m1), and peroxisome proliferator-activated receptor γ (PPARγ; *Pparg*, Mm01184322_m1) was analyzed by quantitative real-time RT-PCR on an ABI Prism 7000 cycler using assays-on-demand kits (TaqMan® Gene Expression Assay, Life Technologies) and normalized to ubiquitin C mRNA (*Ubc*, Mm01198158_m1).

### Liver histology and Oil Red-O staining

Liver tissue was embedded in OCT and samples were cut into 5-μm section, and stained with hematoxylin-eosin. To further evaluate hepatic lipid content with Oil Red O cryosections (7 μm) were fixed in 60% isopropanol for 5 min and stained with 0.5% Oil Red-O (Sigma-Aldrich) in 60% isopropanol for 20 min, and finally counterstained with hematoxylin. Sections were analyzed with standard light microscopy, and relative areas of lipid accumulation (expressed as percentage Oil Red O staining) were quantified using ImageJ software. A minimum of 5 independent fields per sample was evaluated.

### Atherosclerosis quantification

Atherosclerotic plaque formation was determined using en face technique as described [36]. Briefly, thorax of sacrificed mice was opened and aorta was cleaned by removing fat and connective tissue. Then the aorta was excised 2 mm above aortic root and below iliac bifurcation, opened longitudinally and pinned to silicone plates with acupuncture needles (asia-med, Suhl, Germany) and fixed overnight in 4% paraformaldehyde, 5% sucrose, 20 μM EDTA (pH 7.4). Atherosclerotic plaques were stained with Sudan IV for 15 minutes and destained with 75% ethanol. Pictures were taken with a Sony Z-1000 camera and atherosclerotic lesion area was assessed by a person blinded to the samples by using ImageJ software.

### Statistical analyses

Data are given as means ± SEM. Effects of dietary treatment within LDLR^-/-^ mice were analyzed by one-way ANOVA using Dunnett’s multiple comparisons test. Comparisons between LDLR^-/-^ and ApoE^-/-^ mice both fed HFSC were assessed by unpaired two-tailed Student *t* test. A *P* value of ≤0.05 was considered statistically significant.

## Results

### Sucrose-enriched high-fat diet induces earlier insulin resistance and dyslipidemia

**Figure 1:**
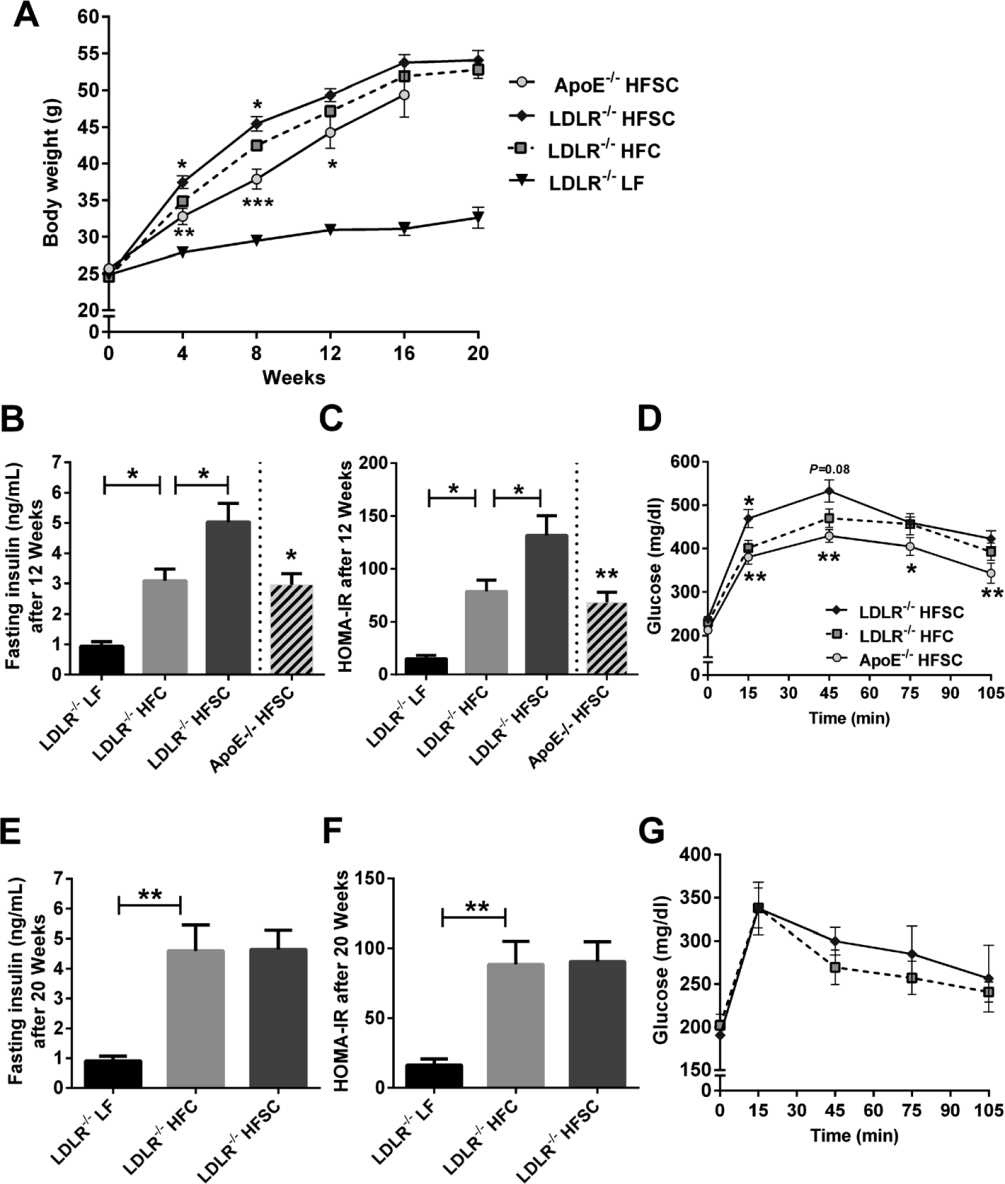
**Sucrose-enriched high-fat diet induces earlier insulin resistance in obese LDLR**^**-/- **^**mice.** LDLR^-/-^ mice were placed on LF control or two different high-fat diets both containing 0.15% cholesterol with (HFSC) or without sucrose enrichment (HFC). ApoE^-/-^ mice were fed HFSC. Mean body weight after dietary treatment up to 20 weeks **(A)**. Fasting plasma insulin and HOMA-IR was determined after treatment with indicated diets for 12 weeks (**B** and **C**; *n* = 8 animals per group except LF-fed LDLR-/- mice with *n* = 6). Glucose tolerance test was performed by intraperitoneal injection of 0.75 g 20% glucose/kg body weight after 12 weeks of feeding (n = 8 animals per group) **(D)**. Fasting plasma insulin **(E)**, HOMA-IR **(F)** and glucose tolerance **(G)** after dietary treatment for 20 weeks (*n* = 5–6 animals per group). For statistical analysis LDLR^-/-^ mice fed HFSC or LF were compared with HFC-fed LDLR^-/-^ mice. In addititon, LDLR^-/-^ and ApoE^-/-^ mice both fed HFSC were compared. All data represent mean ± SEM. *P < 0.05, **P < 0.01, ***P < 0.001.

In order to establish an appropriate mouse model for cardio-metabolic disease, we placed male LDLR^-/-^ mice either on a low-fat control diet (LF) or two different high-fat diets, namely a common high-fat diet containing 0.15% cholesterol (HFC) or a high-fat diet rich in sucrose with 0.15% cholesterol (HFSC). In addition, ApoE^-/-^ mice which were used as a positive control for atherosclerotic plaque formation were fed HFSC. Baseline fasting glucose and insulin concentration before diet start did not significantly differ between LDLR^-/-^ and ApoE^-/-^ mice (Additional file 1: Table S2). LDLR^-/-^ mice fed both HFSC and HFC developed significant diet-induced obesity compared to LF-fed controls with no significant difference between diets (Figure 1A). Also ApoE^-/-^ mice on HFSC showed marked obesity (Figure 1A). Gonadal adipose tissue weight was not significantly altered between HFSC and HFC-fed LDLR^-/-^ mice as well as LDLR^-/-^ compared to ApoE^-/-^ mice both on HFSC (Additional file 2: Figure S1).

After dietary treatment for 12 weeks fasting plasma insulin and HOMA-IR in LDLR^-/-^ mice was higher in the HFSC and HFC group compared to LF. Moreover, HFSC feeding significantly increased fasting insulin level compared to HFC-fed LDLR^-/-^ mice indicating pronounced insulin resistance in animals on HFSC (Figure 1B). Correspondingly, HOMA-IR in LDLR^-/-^ mice on HFSC was significantly increased compared to the HFC-fed group (Figure 1C). ApoE^-/-^ mice developed significantly less pronounced insulin resistance than LDLR^-/-^ mice both on HFSC (Figure 1C). Testing whole body insulin tolerance did not show significant differences between HFSC and HFC group (Additional file 2: Figure S1) while glucose tolerance was impaired in HFSC-fed LDLR^-/-^ mice as illustrated by significantly increased glucose levels in response to a glucose tolerance test at the 15-minute time point (Figure 1D). After 20 weeks on diet, however, there were no differences in fasting insulin levels, HOMA-IR and glucose tolerance in LDLR^-/-^ mice on HFSC and HFC (Figure 1E-G) while body weight did not increase further.

**Figure 2:**
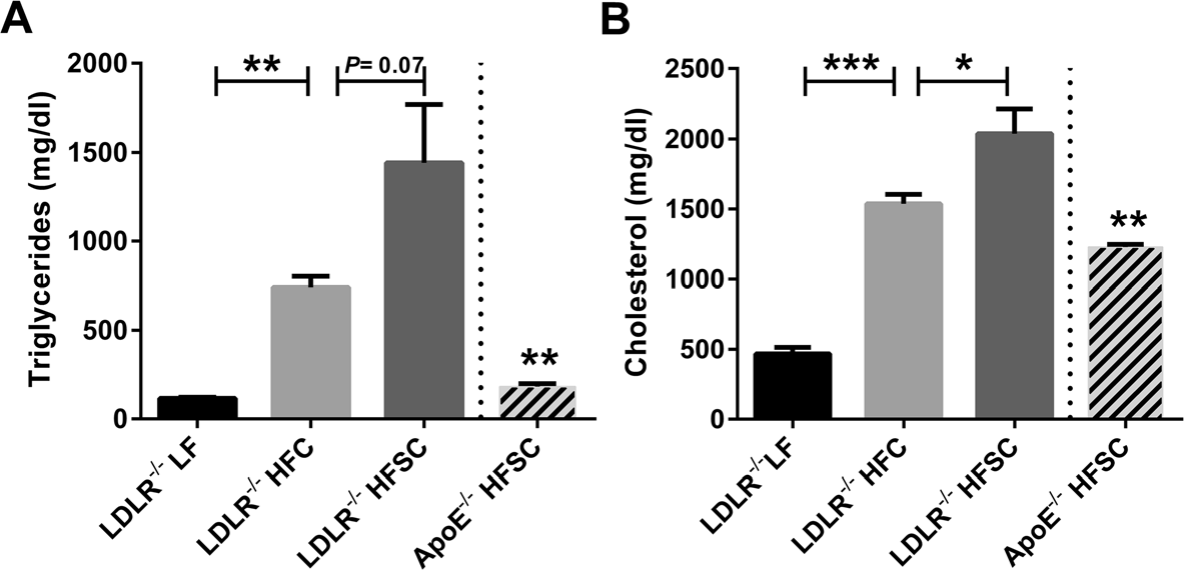
**Effect of dietary treatment on plasma lipids.** Analysis of fasting plasma levels of triglycerides **(A)** and cholesterol **(B)** in LDLR^-/-^ animals fed HFSC, HFC or LF and ApoE^-/-^ mice fed HFSC for 16 weeks (*n* = 5 animals per group). For statistical analysis LDLR^-/-^ mice fed HFSC or LF were compared with HFC-fed LDLR^-/-^ mice. In addititon, LDLR^-/-^ and ApoE^-/-^ mice both fed HFSC were compared. All data represent mean ± SEM. *P < 0.05; **P < 0.01, ***P < 0.001.

Compared to LF-fed LDLR^-/-^ mice, both groups on HFC and HFSC developed hypertriglyceridemia and hypercholesterolemia (Figure 2A,B). Fasting triglyceride levels tended to be higher (*P* = 0.07) and total cholesterol was significantly elevated in LDLR^-/-^ mice on HFSC compared to those on HFC (Figure 2A,B).

**Figure 3:**
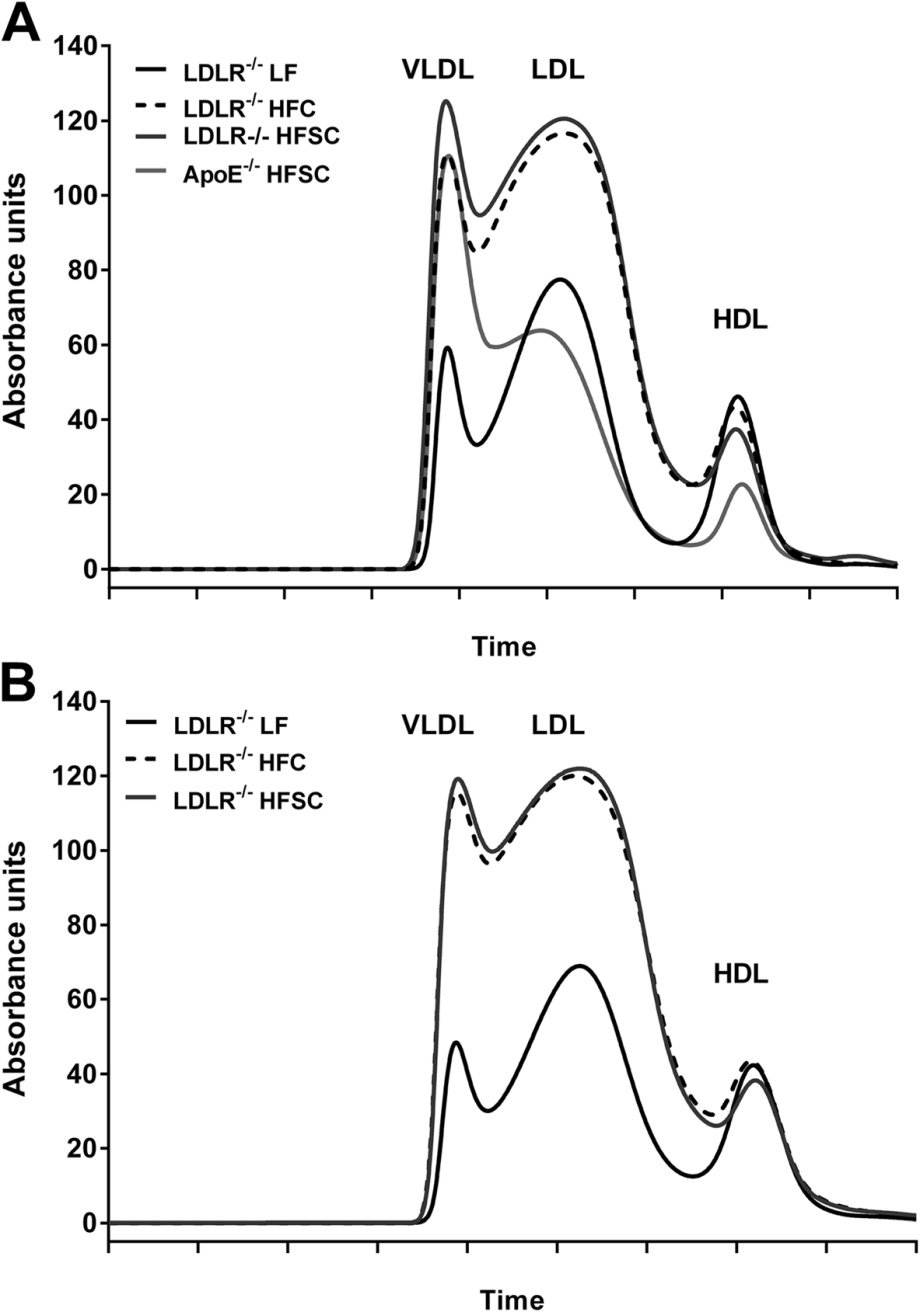
**Analysis of plasma lipoprotein profiles after dietary treatment.** Cholesterol lipoprotein distribution after dietary treatment with indicated diets for 16 weeks **(A)** and 20 weeks **(B)** was analyzed using FPLC (*n* = 5–6 animals per group). All data represent mean.

Fractionation of plasma lipoproteins by FPLC revealed markedly increased cholesterol in VLDL and LDL fractions of obese LDLR^-/-^ mice on both high-fat diets compared to lean LF-fed LDLR^-/-^ mice after 16 and 20 weeks of feeding (Figure 3A,B). Lipoprotein profile of HFSC-fed LDLR^-/-^ mice showed elevated VLDL (*P =* 0.013) and LDL (*P =* 0.04) cholesterol compared to HFC-fed group after 16 but not 20 weeks of diet (Figure 3A,B). In addition, cholesterol in the VLDL and LDL fractions was significantly lower (P ≤ 0.001) in HFSC-fed ApoE^-/-^ mice than HFSC-fed LDLR^-/-^ mice as shown in Figure 3A.

### Sucrose-enrichment of high-fat diet induces more pronounced adipose tissue and hepatic inflammation after 16 weeks of feeding

**Figure 4:**
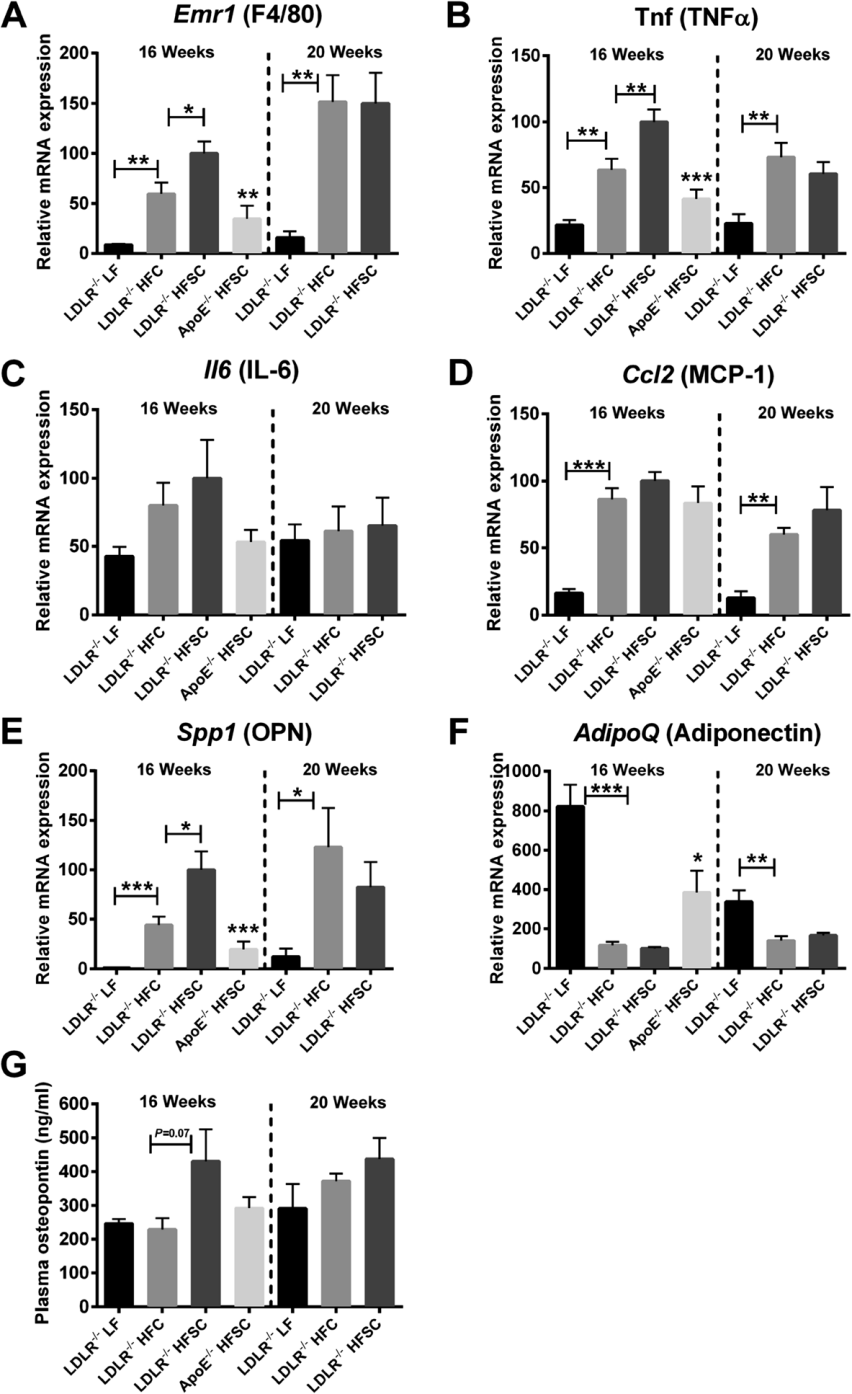
**A sucrose-rich high-fat diet for 16 weeks increases adipose tissue inflammation in obese LDLR**^**-/- **^**mice.** LDLR^-/-^ mice were fed HFSC, HFC or LF for 16 or 20 weeks (*n* = 6 animals per group). ApoE^-/-^ mice were fed HFSC for 16 weeks (*n* = 8 animals per group). Gonadal adipose tissue expression of the genes for macrophage marker F4/80 (encoded by *Emr1*) **(A)**, TNFα (*Tnf*) **(B)**, IL-6 (*Il6*) **(C)**, MCP-1 (*Ccl2*) **(D)**, osteopontin (OPN; *Spp1*) **(E)** and adiponectin (*AdipoQ*) **(F)** was analyzed by real-time RT-PCR. Plasma osteopontin was determined by a commercially available ELISA **(G)**. For statistical analysis LDLR^-/-^ mice fed HFSC or LF were compared with HFC-fed LDLR^-/-^ mice. In addititon, LDLR^-/-^ and ApoE^-/-^ mice both fed HFSC were compared. All data represent mean ± SEM. *P < 0.05, **P < 0.01, ***P < 0.001.

Cytokines secreted by adipose tissue, so-called adipokines, induce local and systemic low-grade inflammation in obesity. In obese adipose tissue macrophages represent the main source of inflammatory adipokines although other cell types, such as lymphocytes, adipocytes, and pre-adipocytes may contribute. To investigate the dietary impact on obesity-associated adipose tissue inflammation in LDLR^-/-^ mice we next analyzed expression of inflammatory adipokines and estimated accumulation of macrophages, T cells and B cells in gonadal adipose tissue. After dietary treatment for 16 weeks mRNA expression of *Emr1*, the gene encoding macrophage marker F4/80, as well as mRNA levels of the inflammatory genes for TNFα (*Tnf*), IL-6 (*Il6*), MCP-1 (*Ccl2*) and osteopontin (*Spp1*) were significantly higher in adipose tissue of obese LDLR^-/-^ mice on both high-fat diets compared to lean control group on LF (Figure 4). In contrast gene expression of the anti-inflammatory and insulin-sensitizing adiponectin (*AdipoQ*) as well as expression of typical genes related to adipocyte function such as those for PPARγ (*Pparg*), FABP4 (*Fabp4*) and fatty acid synthase (FAS, *Fasn*) was significantly lower in obese LDLR^-/-^ mice on both high-fat diets compared to LF-fed group after 16 weeks of feeding (Figure 4F and Additional file 3: Figure S4). Adipose tissue expression of the marker genes specific for pan-T cells (*Cd3e*), cytotoxic (*Cd8a*), and regulatory T cells (*Foxp3*) as well as B cells (*Cd19*) revealed no significant obesity-induced alterations after both high-fat diets compared to lean LF-fed LDLR^-/-^ mice (Additional file 4: Figure S2). In LDLR^-/-^ mice adipose tissue inflammation was significantly higher than in ApoE^-/-^ mice fed the same diet (Figure 4).

Notably, after 16 weeks of feeding adipose tissue inflammation was more pronounced in LDLR^-/-^ mice on HFSC than on HFC as indicated by significantly higher mRNA levels of the genes encoding macrophage marker F4/80, TNF-α, and osteopontin (Figure 4A,B,E). Moreover, the marked increase of osteopontin mRNA levels in adipose tissue was accompanied by a borderline significant increase of osteopontin plasma levels in the HFSC-fed group only (*P* = 0.07), while osteopontin plasma levels in HFC-fed LDLR^-/-^ mice were equal to animals on LF (Figure 4G). After 20 weeks of feeding, all mice showed similar osteopontin plasma levels (Figure 4G).

After 20 weeks on diet, adipose tissue inflammation remained more pronounced in high-fat diet-fed LDLR^-/-^ mice compared to LF while adipose tissue inflammation in the HFSC and HFC group did not remain significantly different from each other suggesting a delayed peak of inflammation in the HFC group (Figure 4).

**Figure 5:**
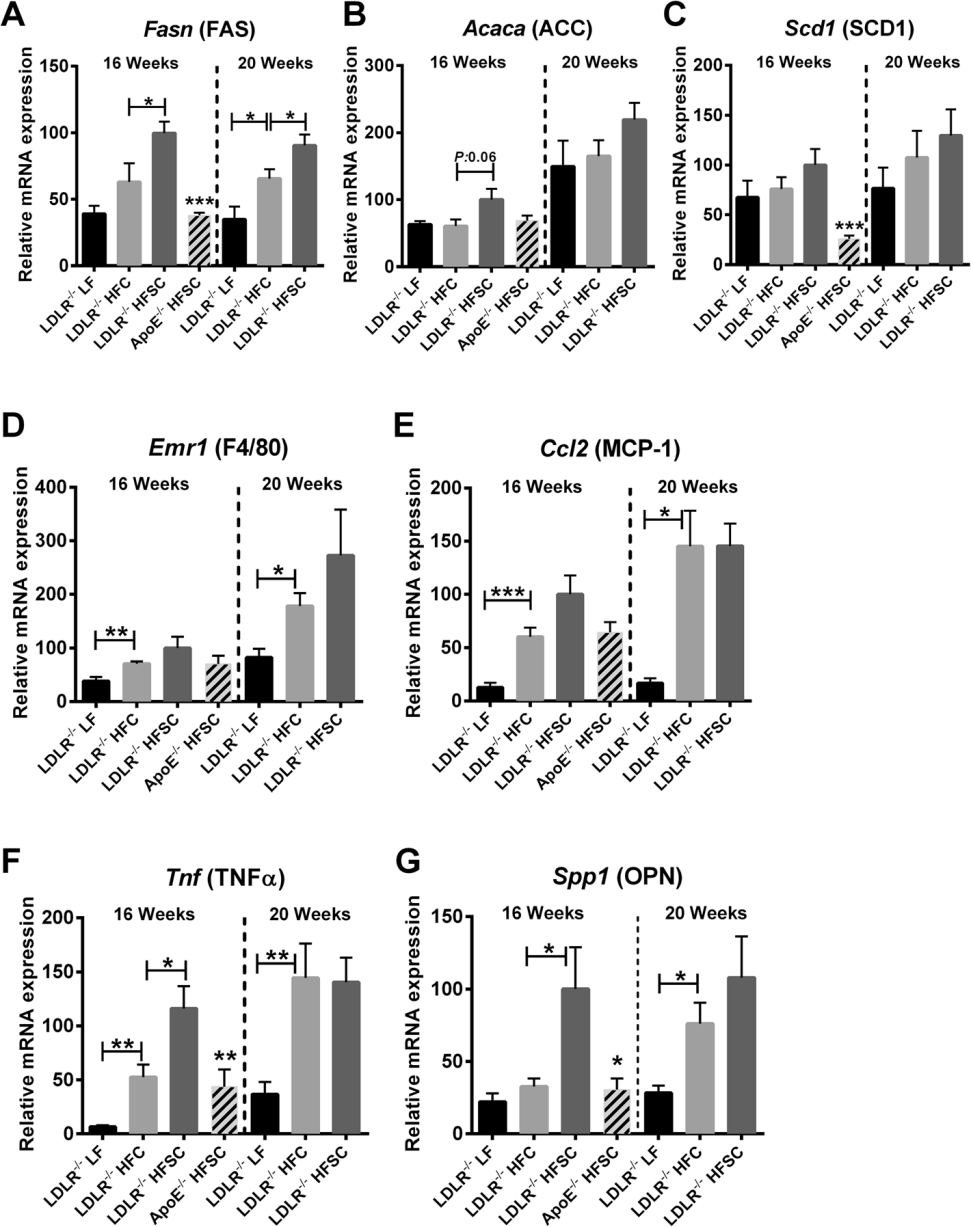
**Sucrose-enriched high fat diet partly accelerates hepatic inflammation.** LDLR^-/-^ mice were fed either HFSC, HFC or LF for 16 or 20 weeks (*n* = 6 animals per group). ApoE^-/-^ mice were fed HFSC for 16 weeks (*n* = 8 animals per group). Hepatic lipogenic gene expression of FAS (*Fas)***(A)**, ACC (*Acaca*) **(B)**, SCD-1 (*Scd1*) **(C)** and hepatic expression of the inflammatory genes for macrophage marker F4/80 (encoded by *Emr1*) **(D)**, MCP-1 (*Ccl2*) **(E)**, TNFα (*Tnf*) **(F)**, and osteopontin (OPN; *Spp1*) **(G)** were analyzed by real-time RT-PCR. For statistical analysis LDLR^-/-^ mice fed HFSC or LF were compared with HFC-fed LDLR^-/-^ mice. In addititon, LDLR^-/-^ and ApoE^-/-^ mice both fed HFSC were compared. All data represent mean ± SEM. *P < 0.05, **P < 0.01, ***P < 0.001.

In order to characterize impact of HFSC compared to HFC feeding on metabolic phenotype in LDLR^-/-^ mice in more detail, we next determined hepatic lipid accumulation and inflammation after treatment with indicated diets. LDLR^-/-^ mice on both high fat diets (HFSC or HFC) developed extensive hepatic steatosis, compared to LF-fed lean controls after 16 as well as 20 weeks of feeding (Additional file 5: Figure S3). Hepatic lipogenic gene expression for FAS (*Fasn*) and acetyl-CoA carboxylase (ACC; *Acaca*) but not stearoyl-CoA desaturase 1 (SCD1; *Scd1*) were elevated in HFSC- compared to HFC-fed LDLR^-/-^ mice after 16 weeks of treatment (Figure 5A-C).

In addition, hepatic inflammation was significantly elevated in obese LDLR^-/-^ mice on both high-fat diets compared to the LF group as shown by increased mRNA expression of the genes for macrophage marker F4/80, MCP-1 and TNFα after 16 weeks of feeding (Figure 5D-F). This high-fat diet-induced increase of hepatic inflammation was even more pronounced after 20 weeks of diet. Of note, HFSC feeding of LDLR^-/-^ mice for 16 weeks induced a significant higher expression of osteopontin and TNFα compared to HFC-fed animals (Figure 5F, G). These differences in inflammatory gene expression evened out after 20 weeks of feeding suggesting that using a sucrose-enriched high-fat diet accelerates some features of fatty liver inflammation.

### Sucrose-enriched high-fat diet accelerates atherosclerotic lesion development

**Figure 6:**
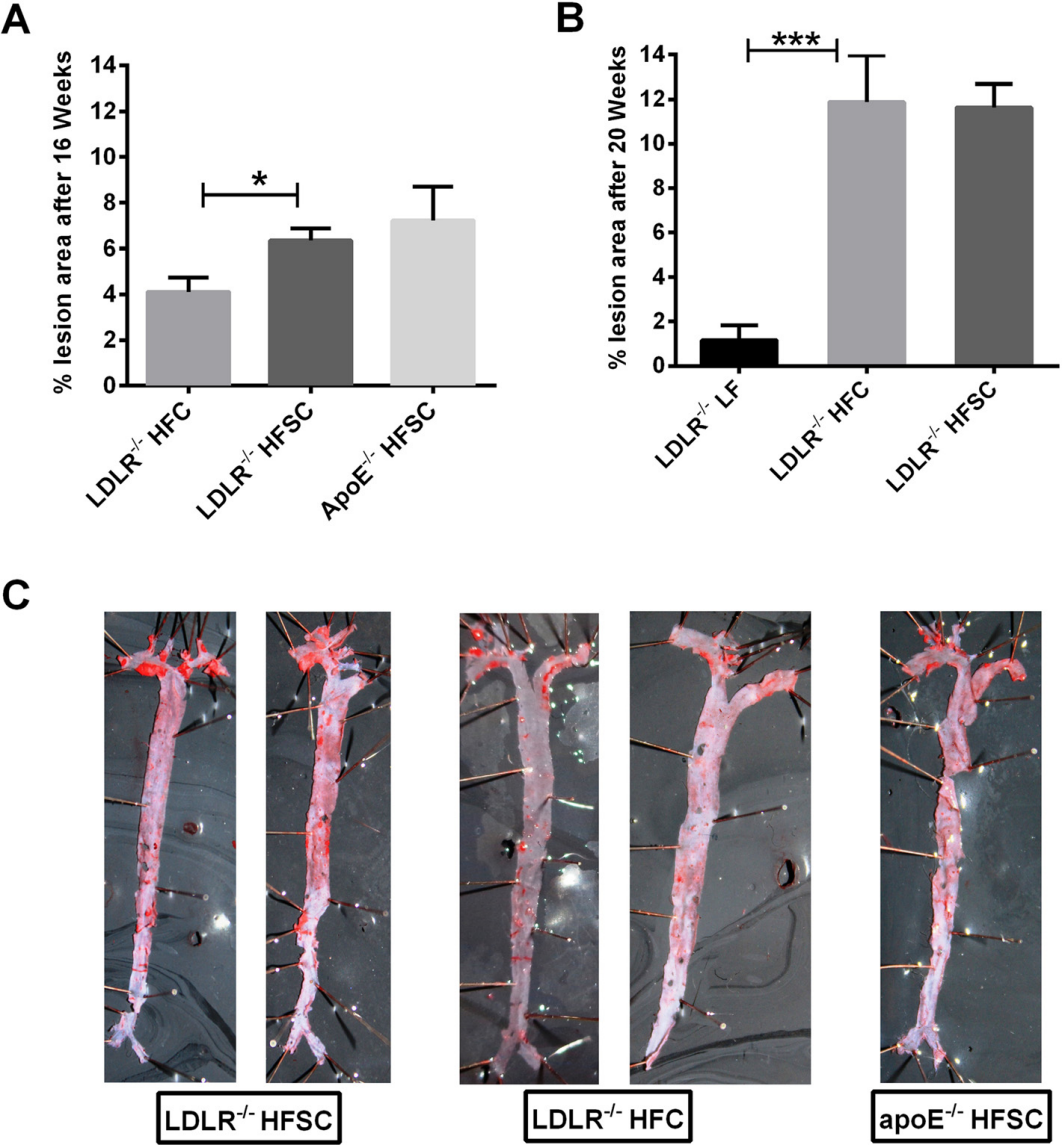
**A sucrose-rich high-fat diet accelerates atherosclerotic plaque formation.** LDLR^-/-^ mice were fed HFSC, HFC or LF and ApoE^-/-^ mice were fed HFSC. Atherosclerotic lesion formation was quantified using en-face analysis after dietary treatment for 16 weeks (**A**; *n* = 5 animals per group) or 20 weeks (**B**; *n* = 6 animals per group). **C**: Representative image of Sudan IV-stained aorta of indicated group after dietary treamtent for 16 weeks. For statistical analysis LDLR^-/-^ mice fed HFSC or LF were compared with HFC-fed LDLR^-/-^ mice. In addititon, LDLR^-/-^ and ApoE^-/-^ mice both fed HFSC were compared. All data represent mean ± SEM. *P < 0.05, ***P < 0.001.

To next determine if HFSC feeding affects atherosclerosis development, atherosclerotic lesion area was quantified in the entire aorta by en face analysis. After 16 weeks of dietary treatment, HFSC-fed LDLR^-/-^ mice achieved similar levels of atherosclerotic lesion area as HFSC-fed ApoE^-/-^ mice which we used as a positive control (Figure 6A). In addition, atherosclerotic lesion formation was significantly increased in LDLR^-/-^ mice fed HFSC compared to LDLR^-/-^ mice fed HFC for 16 weeks (Figure 6A,C). Only after 20 weeks of dietary treatment, LDLR^-/-^ mice on HFSC and HFC achieved similar levels of atherosclerotic lesion areas (Figure 6B).

## Discussion

Obesity and particularly the metabolic syndrome are associated with both, type 2 diabetes based on insulin resistance and cardiovascular disease. Obesity induces adipose tissue inflammation, which plays a key role in the development of obesity-driven metabolic complications leading the way to type 2 diabetes and cardiovascular diseases [2,5]. Our data show and confirm that both LDLR^-/-^ and ApoE^-/-^ mice develop diet-induced obesity on high-fat diets, while LDLR^-/-^ mice are more prone to adipose tissue inflammation and insulin resistance in addition to atherosclerosis. Hence, this genetic background turned out to be more suitable for investigation of cardio-metabolic disease. Moreover, we show here that using sucrose rather than more complex carbohydrates in a high-fat diet significantly accelerated the development of obesity-induced adipose tissue inflammation, insulin resistance, impaired glucose tolerance and atherosclerosis in LDLR^-/-^ mice leading to suitable readouts already after 12 to 16 weeks of dietary treatment.

After dietary treatment of LDLR^-/-^ mice with HFSC or HFC for 16 weeks mice on HFSC revealed significantly more pronounced adipose tissue inflammation as shown by elevated adipose tissue expression of the inflammatory genes for F4/80, osteopontin and TNF-α Increased gene expression of F4/80, a typical macrophage marker, indicates macrophage accumulation, while increased osteopontin and TNF-αm RNA levels provide additional evidence for local inflammation. Strikingly, after four more weeks, adipose tissue inflammation in LDLR^-/-^ mice fed HFSC or HFC reached similar levels of inflammatory gene expression suggesting that the high sucrose uptake speeds up rather than augments the detrimental effects of high-fat and cholesterol-rich diets. In addition to adipose tissue inflammation, LDLR^-/-^ mice fed HFSC further demonstrated increased hepatic inflammation compared to HFC-fed LDLR^-/-^ mice after 16 weeks of feeding while these differences evened out after 20 weeks. These results may indicate an important role of dietary macronutrient composition e.g. on inflammatory alterations which grossly exceeds its effect on body weight [37-39].

At earlier time points, we observed marked differences in adipose tissue inflammation, insulin levels, HOMA-IR, and glucose tolerance between the LDLR^-/-^ animals on HFSC and those on HFC while the insulin tolerance test did not show significant differences suggesting that whole body insulin resistance was not affected by HFSC. Strikingly, after 20 weeks on diet, the stabilization of adipose tissue inflammation was mirrored by comparable levels of fasting insulin and HOMA-IR, which on the one hand might support the notion that adipose tissue inflammation contributes to insulin resistance [6,40]. On the other hand, this observation could be due to the fact that after a certain time span of feeding maximal biological effects might be reached in HFSC-fed LDLR^-/-^ mice and thus reach a plateau while HFC-fed LDLR^-/-^ mice then “catch up” with the HFSC-fed group. In general this might be an issue of many experimental diets which are often very extreme in their composition to reach maximum biological effects very fast. Moreover, although body weight after dietary treatment of LDLR^-/-^ mice with HFSC or HFC for 12 weeks did not differ, total body weight exposure (area under the body weight curve) was moderately higher in HFSC- compared to HFC-fed group (LDLR^-/-^ mice on HFSC: 807 ± 23 and HFC: 737 ± 14), which could also contribute to difference in metabolic parameters at this time point.

Notably, mice on both diets used in this study appeared healthy at any time point, which is an advantage over the widely used Western Diets which reportedly leads to changes in fur and skin integrity even leading to ulceration or sudden weight loss [41].

After 16 weeks of diet LDLR^-/-^ mice on HFSC but not on HFC developed atherosclerotic lesion areas comparable to the commonly used ApoE^-/-^ model. The significant early increase of total plasma cholesterol as well as an increase of VLDL and LDL cholesterol levels in HFSC-fed LDLR^-/-^ mice may certainly contribute to plaque formation and represent major risk factors for atherosclerosis [42]. On the other hand, the marked adipose tissue inflammation and insulin resistance in HFSC-fed animals might further suggest an association between obesity-induced inflammation and atherosclerotic plaque formation which was described in murine models [43] and is evident in clinical trials [44,45].

In addition to increased osteopontin mRNA levels in adipose tissue and liver, plasma osteopontin was in trend upregulated by the sucrose-enriched high-fat diet (HFSC; *P* = 0.077), but not the common high-fat diet (HFC) confirming earlier results that high-fat diet locally upregulates osteopontin in tissue but not in plasma [46]. HFSC-induced elevated plasma osteopontin could be directly related to its upregulation by glucose, which was shown in vascular smooth muscle cells and arteries of diabetic patients [47,48]. Thus, osteopontin seems to primarily act locally and is closely related to atherosclerotic disease and cardiovascular events in patients [49].

In humans it is well known that diets with a high dietary glycemic load lead to inflammation, insulin resistance and impaired beta-cell function [20,22] and promote weight gain [50]. Of course one has to bear in mind that mice used in this study were of C57BL/6 background which is known to have smaller islet mass and more prominent islet inflammation than other mouse strains, [51] but C57BL/6 are a widely accepted mouse model for obesity-associated complications.

Here we show that in LDLR^-/-^ mice addition of sucrose to a high-fat diet induces earlier and more pronounced adipose tissue inflammation, insulin resistance, glucose intolerance and atherosclerosis. As sucrose consists of both fructose and glucose our findings of worsened cardio-metabolic situation in LDLR^-/-^ mice fed HFSC may be due to the high glycemic load on the one hand and fructose-induced adverse effects on the other hand. E.g. sucrose could partly contribute to accelerated inflammation through fructose, which has been shown to activate inflammatory pathways such as NF-κB signaling and to induce oxidative stress in animal models which might further contribute to inflammatory alterations [52-54]. Moreover, fructose in high amounts has been shown to decrease insulin sensitivity more than glucose and to induce ectopic and visceral fat deposition, *de novo* lipogenesis, higher blood pressure and blood uric acid concentrations [23,55]. The fact that fructose absorption is enhanced in presence of glucose [56], may explain why fructose-enriched diets alone could not induce insulin resistance in LDLR^-/-^ mice [57].

## Conclusions

In conclusion, our study demonstrates that feeding LDLR^-/-^ mice a sucrose-rich high-fat diet (HFSC) constitutes a fast and suitable animal model to investigate the pathogenesis of cardio-metabolic disease including adipose tissue inflammation, insulin resistance and atherosclerosis. Saving a couple of weeks in every animal experiment will provide a significant advantage in everyday research. In addition, our data point to the close interrelation between adipose tissue inflammation, insulin resistance and atherosclerosis.

## Abbreviations

ACC: Acetyl-CoA carboxylase; ApoE-/- mice: Apolipoprotein E-deficient mice; FABP4: Fatty acid binding protein 4; HFC: High-fat diet supplemented with 0.15% cholesterol; HFSC: High-fat, high-sucrose diet supplemented with 0.15% cholesterol; HOMA-IR: Homeostasis model assessment of insulin resistance; IL-6: Interleukin 6; LDLR-/- mice: LDL receptor-deficient mice; LF: Low-fat control diet; MCP-1: Monocyte chemoattractant protein-1; OPN: Osteopontin; PPARγ: Peroxisome proliferator-activated receptor γ; SCD1: Stearoyl-CoA desaturase 1; TNFα: Tumor necrosis factor-α.

## Competing interests

The authors declare that they have no competing interests.

## Supplementary Material

Additional file 1: Table S1 Composition of the used diets. **Table S2:** Baseline characteristics. Click here for file

Additional file 2: Figure S1 LDLR-/- mice were placed on LF control or two different high-fat diets both containing 0.15% cholesterol with (HFSC) or without sucrose enrichment (HFC). ApoE-/- mice were fed HFSC. Mean gonadal adipose tissue (GWAT) weight after dietary treatment for 16 and 20 weeks (A, B). Insulin tolerance test was performed by intraperitoneal injection of 0.75 g insulin/kg body weight after 12 and 20 weeks of feeding. Asterisk indicates significant difference between ApoE-/- and LDLR-/- mice on HFSC (n = 8 animals per group) (C, D). For statistical analysis LDLR-/- mice fed HFSC or LF were compared with HFC-fed LDLR-/- mice. In addititon, LDLR-/- and ApoE-/- mice both fed HFSC were compared. All data represent mean ± SEM. *P < 0.05, **P < 0.01, ***P < 0.001. Click here for file

Additional file 3: Figure S4 LDLR-/- mice were fed HFSC, HFC or LF for 16 or 20 weeks (*n* = 6 animals per group). ApoE-/- mice were fed HFSC for 16 weeks (*n* = 8 animals per group). Gonadal adipose tissue expression of the genes for PPARγ (*Pparg*) (A), FABP4 (*Fabp4*) (B), and fatty acid synthase (FAS; *Fasn*) (C), and Leptin (*Lep*) (D) was determined by real-time RT-PCR. For statistical analysis LDLR-/- mice fed HFSC or LF were compared with HFC-fed LDLR-/- mice. In addititon, LDLR-/- and ApoE-/- mice both fed HFSC were compared. All data represent mean ± SEM. *P < 0.05, **P < 0.01, ***P < 0.001. Click here for file

Additional file 4: Figure S2 LDLR-/- mice were fed HFSC, HFC or LF for 16 or 20 weeks (*n* = 6 animals per group). ApoE-/- mice were fed HFSC for 16 weeks (*n* = 8 animals per group). Gonadal adipose tissue expression of the marker genes for pan-T cells (*Cd3e*) (A), cytotoxic (*Cd8a*) (B), and regulatory T cells (*Foxp3*) (C) as well as B cells (*Cd19*) (D) was analyzed by real-time RT-PCR. For statistical analysis LDLR-/- mice fed HFSC or LF were compared with HFC-fed LDLR-/- mice. In addititon, LDLR-/- and ApoE-/- mice both fed HFSC were compared. All data represent mean ± SEM. Click here for file

Additional file 5: Figure S3 LDLR^-/-^ mice were fed either HFSC, HFC or LF for 16 or 20 weeks and ApoE^-/-^ mice were fed HFSC for 16 weeks. Hematoxylin-esoin and Oil Red O staining of liver sections was performed (*n* = 4-5 animals per group). Representative pictures after dietary treatment for 16 (A) or 20 weeks (B) are given in 20-fold magnification. Quantification of lipid accumulation in Oil Red O-stained sections (C). For statistical analysis LDLR^-/-^ mice fed HFSC or LF were compared with HFC-fed LDLR^-/-^ mice. In addititon, LDLR^-/-^ and ApoE^-/-^ mice both fed HFSC were compared. All data represent mean ± SEM. Click here for file
