# Supplementary material for: An accelerated mouse model for atherosclerosis and adipose tissue inflammation
Source: Cardiovasc Diabetol. 2014 Jan 17;13:23. doi: 10.1186/1475-2840-13-23 (PMC3902066; doi:10.1186/1475-2840-13-23)
Supplement: Additional file 1: Table S1 — Composition of the used diets. Table S2: Baseline characteristics. [file 1475-2840-13-23-S1.pdf]

Additional Table 1: Composition of the used diets

Composition	Diets		
	**LF**	**HFC**	**HFSC**
**Kcal/g**	3.85	5.24	5.54
**Carbohydrate (kcal%)**	70	20	28
**Sucrose (kcal%)**	34.5	6.8	17.5
**Fat (kcal%)**	10	59.9	58
**Protein (kcal%)**	20	20	15
**Carbohydrate (wt%)**	67.3	26.3	38.1
**Fat (wt%)**	4.3	34.8	35.5
**Protein (wt%)**	19.2	26.2	20.5
**Sucrose (g/kg)**	331.7	88.8	241.6
**Maltodextrin 10 (g/kg)**	33.2	161.3	128.5
**Corn Starch (g/kg)**	298.6	0	0
**Lard* (g/kg)**	19	316.1	354.6
**Soybean Oil (g/kg)**	23.7	32.3	0
**Casein* (g/kg)**	189.6	258.1	201.7
**Cholesterol added (wt%)**	0	0.15	0.15

All diets also contained: vitamine and mineral mix.

*Lard and Casein add small amounts of cholesterol: Used estimates of cholesterol content in lard = 0.72 mg per g and in Casein = 0.2 mg per gram (Product Data Sheet; Research Diets).

Additional Table 2: Baseline characteristics

Parameter	$LDLR^{-/-}$ mice	$ApoE^{-/-}$ mice
Body weight (g)	23..5 ± 0.3	24.2 ± 0.5
Fasting Glucose (mg/dL)	187 ± 7	179 ± 9
Fasting Insulin (ng/mL)	0.79 ± 0.08	1.06 ± 0.12

Fasting glucose was measured in total blood samples and insulin was analyzed in plasma (*n*=6-8 animals per group). Results are expressed as mean ± SEM. There were no statistically significant differences between the LDLR-/- and ApoE-/- mice for all analyzed parameters.
