# Supplementary material for: An accelerated mouse model for atherosclerosis and adipose tissue inflammation
Source: Cardiovasc Diabetol. 2014 Jan 17;13:23. doi: 10.1186/1475-2840-13-23 (PMC3902066; doi:10.1186/1475-2840-13-23)
Supplement: Additional file 2: Figure S1 — LDLR-/- mice were placed on LF control or two different high-fat diets both containing 0.15% cholesterol with (HFSC) or without sucrose enrichment (HFC). ApoE-/- mice were fed HFSC. Mean gonadal adipose tissue (GWAT) weight after dietary treatment for 16 and 20 weeks (A, B). Insulin tolerance test was performed by intraperitoneal injection of 0.75 g insulin/kg body weight after 12 and 20 weeks of feeding. Asterisk indicates significant difference between ApoE-/- and LDLR-/- mice on HFSC (n = 8 animals per group) (C, D). For statistical analysis LDLR-/- mice fed HFSC or LF were compared with HFC-fed LDLR-/- mice. In addititon, LDLR-/- and ApoE-/- mice both fed HFSC were compared. All data represent mean ± SEM. *P < 0.05, **P < 0.01, ***P < 0.001. [file 1475-2840-13-23-S2.pdf]

Additional Figure 1

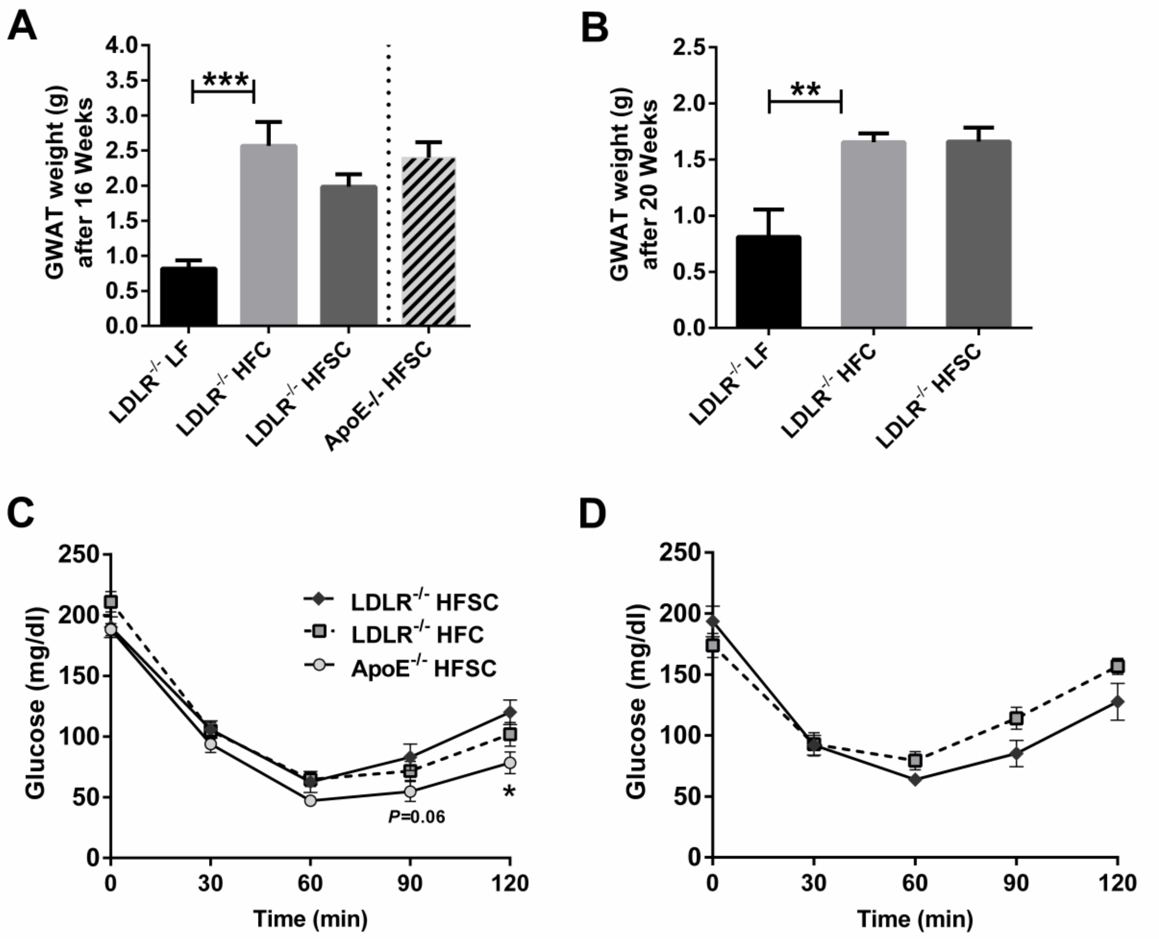

Additional Figure 1: LDLR$^{-/-}$ mice were placed on LF control or two different high-fat diets both containing 0.15% cholesterol with (HFSC) or without sucrose enrichment (HFC). ApoE$^{-/-}$ mice were fed HFSC. Mean gonadal adipose tissue (GWAT) weight after dietary treatment for 16 and 20 weeks (A,B). Insulin tolerance test was performed by intraperitoneal injection of 0.75 g insulin/kg body weight after 12 and 20 weeks of feeding. Asterisk indicates significant difference between ApoE$^{-/-}$ and LDLR$^{-/-}$ mice on HFSC (n = 8 animals per group) (C,D). For statistical analysis LDLR$^{-/-}$ mice fed HFSC or LF were compared with HFC-fed LDLR$^{-/-}$ mice. In addititon, LDLR$^{-/-}$ and ApoE$^{-/-}$ mice both fed HFSC were compared. All data represent mean ± SEM. *P<0.05, **P<0.01, ***P<0.001
