# Supplementary material for: An accelerated mouse model for atherosclerosis and adipose tissue inflammation
Source: Cardiovasc Diabetol. 2014 Jan 17;13:23. doi: 10.1186/1475-2840-13-23 (PMC3902066; doi:10.1186/1475-2840-13-23)
Supplement: Additional file 3: Figure S4 — LDLR-/- mice were fed HFSC, HFC or LF for 16 or 20 weeks (n = 6 animals per group). ApoE-/- mice were fed HFSC for 16 weeks (n = 8 animals per group). Gonadal adipose tissue expression of the genes for PPARγ (Pparg) (A), FABP4 (Fabp4) (B), and fatty acid synthase (FAS; Fasn) (C), and Leptin (Lep) (D) was determined by real-time RT-PCR. For statistical analysis LDLR-/- mice fed HFSC or LF were compared with HFC-fed LDLR-/- mice. In addititon, LDLR-/- and ApoE-/- mice both fed HFSC were compared. All data represent mean ± SEM. *P < 0.05, **P < 0.01, ***P < 0.001. [file 1475-2840-13-23-S3.pdf]

Additional Figure 4

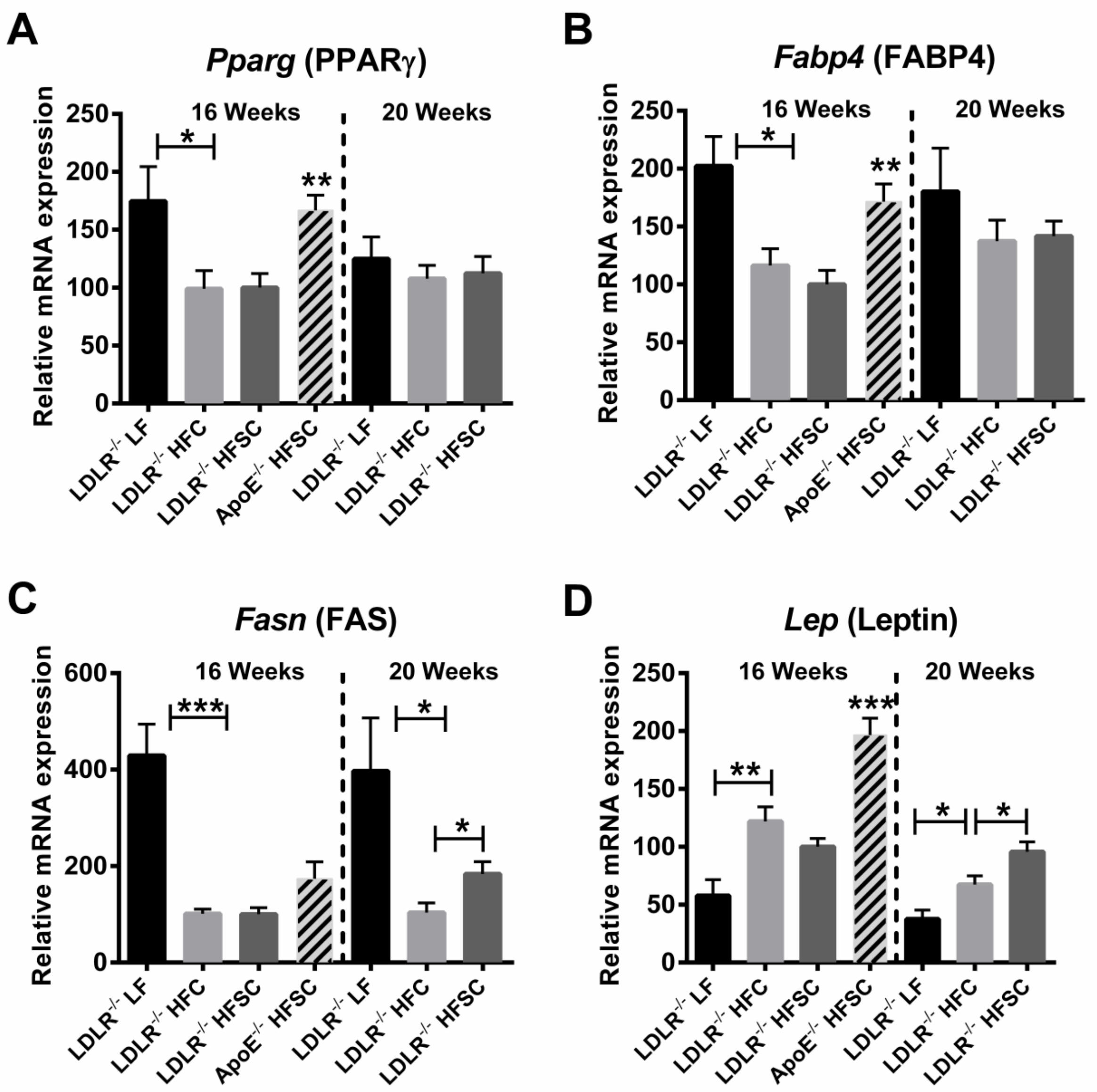

Additional Figure 4: LDLR$^{-/-}$ mice were fed HFSC, HFC or LF for 16 weeks or 20 weeks (*n* = 6 animals per group). ApoE$^{-/-}$ mice were fed HFSC for 16 weeks (*n* = 8 animals per group). Gonadal adipose tissue expression of the genes for PPARγ (*Pparg*) (A), FABP4 (*Fabp4*) (B), and fatty acid synthase (FAS; *Fasn*) (C), and Leptin (*Lep*) (D) was determined by real-time RT-PCR. For statistical analysis LDLR$^{-/-}$ mice fed HFSC or LF were compared with HFC-fed LDLR$^{-/-}$ mice. In addititon, LDLR$^{-/-}$ and ApoE$^{-/-}$ mice both fed HFSC were compared. All data represent mean ± SEM. *P<0.05, **P<0.01, ***P<0.001
