# Supplementary material for: An accelerated mouse model for atherosclerosis and adipose tissue inflammation
Source: Cardiovasc Diabetol. 2014 Jan 17;13:23. doi: 10.1186/1475-2840-13-23 (PMC3902066; doi:10.1186/1475-2840-13-23)
Supplement: Additional file 4: Figure S2 — LDLR-/- mice were fed HFSC, HFC or LF for 16 or 20 weeks (n = 6 animals per group). ApoE-/- mice were fed HFSC for 16 weeks (n = 8 animals per group). Gonadal adipose tissue expression of the marker genes for pan-T cells (Cd3e) (A), cytotoxic (Cd8a) (B), and regulatory T cells (Foxp3) (C) as well as B cells (Cd19) (D) was analyzed by real-time RT-PCR. For statistical analysis LDLR-/- mice fed HFSC or LF were compared with HFC-fed LDLR-/- mice. In addititon, LDLR-/- and ApoE-/- mice both fed HFSC were compared. All data represent mean ± SEM. [file 1475-2840-13-23-S4.pdf]

Additional Figure 2

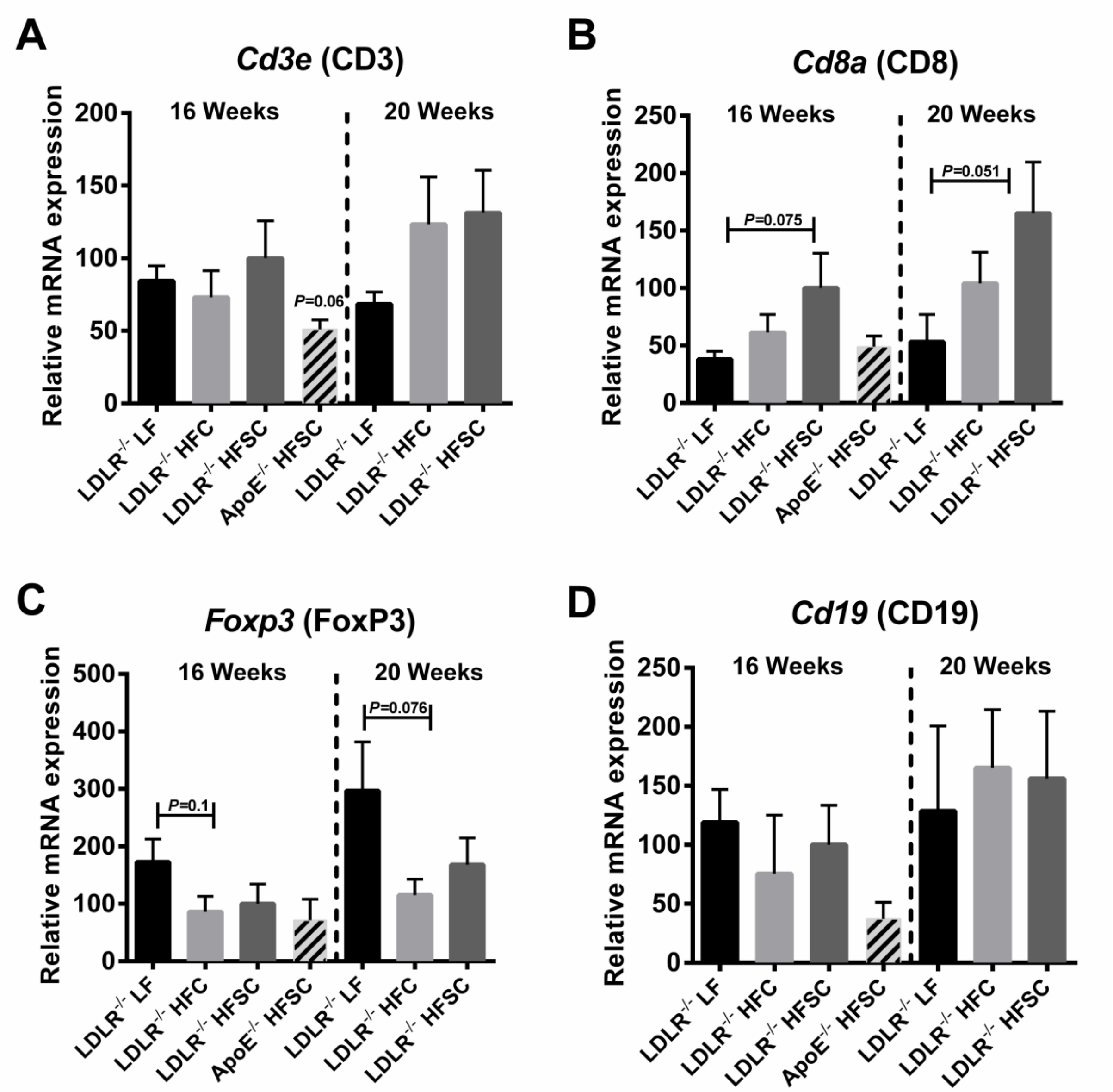

Additional Figure 2: LDLR$^{-/-}$ mice were fed HFSC, HFC or LF for 16 weeks or 20 weeks (n = 6 animals per group). ApoE$^{-/-}$ mice were fed HFSC for 16 weeks (n = 8 animals per group). Gonadal adipose tissue expression of the marker genes for pan-T cells (*Cd3e*) (A), cytotoxic (*Cd8a*) (B), and regulatory T cells (*Foxp3*) (C) as well as B cells (*Cd19*) (D) was analyzed by real-time RT-PCR. For statistical analysis LDLR$^{-/-}$ mice fed HFSC or LF were compared with HFC-fed LDLR$^{-/-}$ mice. In addititon, LDLR$^{-/-}$ and ApoE$^{-/-}$ mice both fed HFSC were compared. All data represent mean ± SEM.
