# Supplementary material for: An accelerated mouse model for atherosclerosis and adipose tissue inflammation
Source: Cardiovasc Diabetol. 2014 Jan 17;13:23. doi: 10.1186/1475-2840-13-23 (PMC3902066; doi:10.1186/1475-2840-13-23)
Supplement: Additional file 5: Figure S3 — LDLR-/- mice were fed either HFSC, HFC or LF for 16 or 20 weeks and ApoE-/- mice were fed HFSC for 16 weeks. Hematoxylin-esoin and Oil Red O staining of liver sections was performed (n = 4-5 animals per group). Representative pictures after dietary treatment for 16 (A) or 20 weeks (B) are given in 20-fold magnification. Quantification of lipid accumulation in Oil Red O-stained sections (C). For statistical analysis LDLR-/- mice fed HFSC or LF were compared with HFC-fed LDLR-/- mice. In addititon, LDLR-/- and ApoE-/- mice both fed HFSC were compared. All data represent mean ± SEM. [file 1475-2840-13-23-S5.doc]

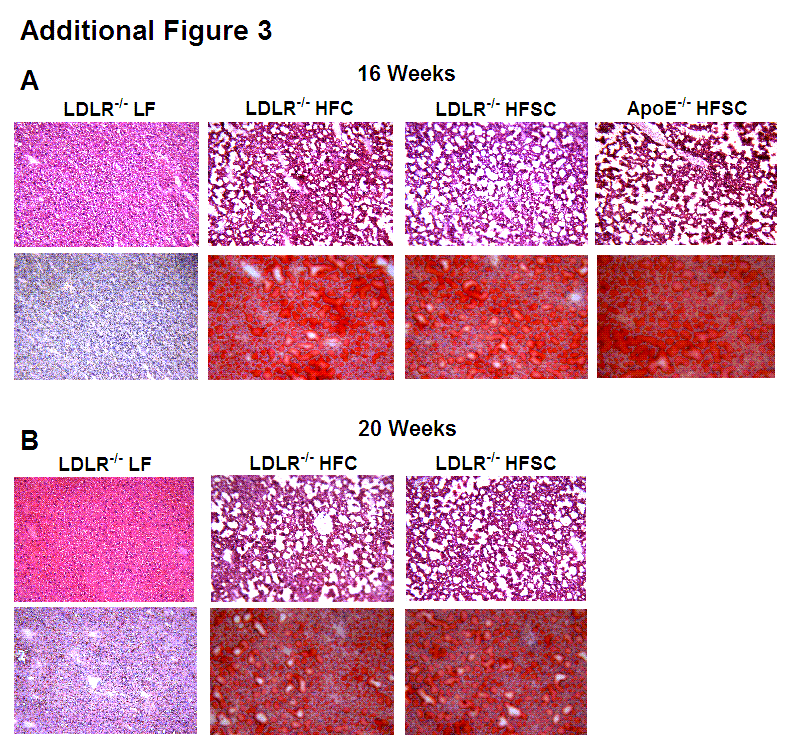

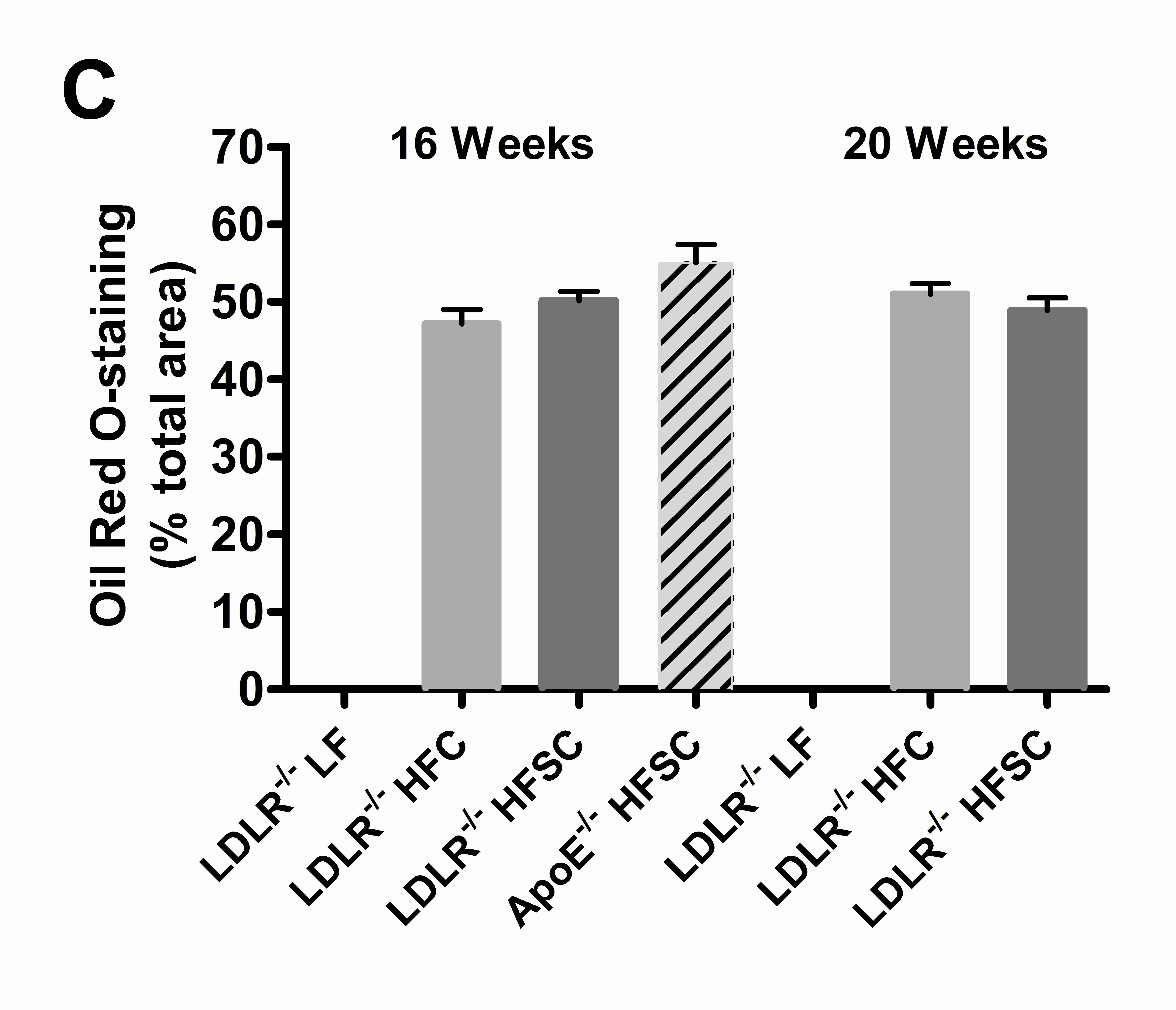


**Additional Figure 3:** LDLR^-/-^ mice were fed either HFSC, HFC or LF for 16 or 20 weeks and ApoE^-/-^ mice were fed HFSC for 16 weeks (*n* = 8 animals per group). Hematoxylin-esoin and Oil Red O staining of liver sections was performed (*n* = 4-5 animals per group). Representative pictures after dietary treatment for 16 (A) or 20 weeks (B) are given in 20-fold magnification. Quantification of lipid accumulation in Oil Red O-stained sections (C). For statistical analysis LDLR^-/-^ mice fed HFSC or LF were compared with HFC-fed LDLR^-/-^ mice. In addititon, LDLR^-/-^ and apoE^-/-^ mice both fed HFSC were compared. All data represent mean ± SEM.
